# Meat quality of pork loins from Hereford×Berkshire female and intact male pigs reared in an alternative production system

**DOI:** 10.5713/ajas.18.0670

**Published:** 2019-02-07

**Authors:** Yvette Robbins, Hyeon-Suk Park, Travis Tennant, Dana Hanson, Niki Whitley, Byungrok Min, Sang-Hyon Oh

**Affiliations:** 1 Department of Animal Science, North Carolina Agricultural and Technical State University, Greensboro, NC 27411, USA; 2 Department of Agricultural Sciences, West Texas A&M University, Canyon, TX 79016, USA; 3 Department of Food, Bioprocessing, & Nutrition Sciences, North Carolina State University, Raleigh, NC 27695, USA; 4 Department of Agricultural Sciences, Fort Valley State University, Fort Valley, GA 31030, USA; 5 Department of Agriculture, Food, and Resource Sciences, University of Maryland Eastern Shore, Princess Ann, MD 21853, USA

**Keywords:** Pork Quality, Boar Taint, Outdoor Production, Sex

## Abstract

**Objective:**

The objective of the present study was to investigate pork quality from Hereford× Berkshire female and intact male pigs reared outdoors in an alternative production system.

**Methods:**

Berkshire purebred sows were artificially inseminated, once in the fall and again in the spring of the following year, with semen from Hereford boars and managed free of antibiotics in an outdoor hoop structure until the last month of pregnancy, after which they were moved to a pasture-based unit of 0.8 hectares with individual lots with a farrowing hut, shade, and water *ad libitum*. Piglets were weaned at 4 weeks of age and housed in a deep-bedded hoop structure, grouped by sex. Animals were harvested at market weight of 125 kg, approximately 200 days of age. Hot carcass weight was collected at the time of the harvest. After 24 hours of refrigeration, carcass characteristics were measured. *Longissimus dorsi* samples collected from the right side loin. Loins were cut into 2.54-cm thick chops and were used to measure marbling score, color score, drip loss, and ultimate pH. Sensory panel tests were conducted as well at North Carolina State University. For pork characteristics and sensory panel data, trial and sex were included in the statistical model as fixed effects. Hot carcass weight was included in the model as a covariate for backfat thickness.

**Results:**

Neither the subjective nor the objective color scores displayed any differences between the boars and the gilts. No difference was found for pH and marbling score between trials or sexes. Gilts had a thicker backfat measurement at the last lumbar and a narrower longissimus muscle area measurement when compared to the boars. The only difference in the sensory characteristics was found between the trials for texture and moisture scores.

**Conclusion:**

Consumers were not able to detect boar taint under the condition of this study, which is that the intact males were reared outdoors. Additional trials would be necessary; however, based on the results of the present study, outdoor rearing can be suggested as a solution to the issue of boar taint.

## INTRODUCTION

Modern day definition of meat quality has evolved from an objective assessment obtained through quantitative performance tests to include a subject assessment shaped by nonquantitative factors, such as rearing condition, use of antibiotics or unnatural surgical procedures, and etc., in response to the growing consumer concerns about animal welfare, which have led to a higher preference for products from organically raised animals. Among modern day consumers, a perceived gap exists between the conventional and organic pig production methods [1]. Generally, consumers view pigs raised in organic production systems as happy and naturally raised, without growth promoters, given larger spaces and allowed to exercise natural behaviors. Conversely, consumers view pigs raised in conventional systems as unhappy, unable to roam freely as they would in nature, and unnaturally grown at a faster rate. Consumers also associate production method with pork quality [2]. When products were labeled “organic” or “free range”, consumers applied positive attributes, such as better animal welfare and tastiness, to the products [3]. Furthermore, when information about the process characteristics of the product was provided, consumers were willing to pay premium for the products labeled organic or natural [4].

In response to growing concerns of consumers, production systems in which animals are naturally or organically raised have grown in popularity among producers. One of the characteristics that define an organic farming system is the absence of surgical procedures, such as the castration of the boars. Though the absence of castration appeals to the consumers for its consideration of animal welfare, it is known to depreciate the product value due to the negative eating quality associated with the meat. The negative eating quality, namely the foul smell in the cooked meat referred to as boar taint, is caused by three main biochemical compounds: Androstenone, skatole, and indole [5,6]. Breed difference in boar taint score, based on the amount of the three main biochemical compounds in the meat, have been identified by a number of studies [7–9], and heritability of the boar taint compounds makes it possible to selectively breed against them [10]. Furthermore, evidence suggests there is no difference in the eating qualities of gilts and uncastrated boars when the animals are reared outdoors [11,12]. Therefore, the objective of the present study was to investigate the eating quality of pork from Hereford×Berkshire (HB) female and intact male pigs reared outdoors in an alternative production system. It was hypothesized that there would not be any difference in meat qualities between uncastrated male pigs and female pigs. The system was not labeled organic due to the lack of certification of the facility; however, the rearing environment was kept as close to a rearing environment at a certified organic production facility as possible.

## MATERIALS AND METHODS

### Animals

The experiment was conducted at NC A&T State University, located in Greensboro, NC, USA (IACUC: 12-003.0). Two trials were conducted, once in the fall of 2015 and once in the spring of 2016. The sows used for breeding were Berkshire purebreds artificially inseminated with semen from Hereford boars and managed free of antibiotics in an outdoor hoop structure until the last month of pregnancy, after which they were moved to a pasture-based unit of 0.8 hectares with individual lots (14×24 m^2^) with a farrowing hut, shade, and water *ad libitum*. Piglets were weaned at 4 weeks of age and housed in a deep-bedded hoop structure, grouped by sex. In the first trial, there were 8 females and 13 intact males. In the second trial, there were 5 females and 5 intact males. The piglets were given access to standard National Research Council balanced rations and water *ad libitum*. Variables measured are listed in Table 1.

### Carcass data collection

In the first trial, 6 Berkshire sows sired by Hereford farrowed 21 HB pigs. In the second trial, 3 sows farrowed 10 HB pigs. All of the females from the first and the second trials were used to collect carcass data. Of the 13 intact males in the first trial, 8 were randomly selected to match the number of females in the first trial. All of the intact males in the second trial were used. Animals were harvested at a USDA-inspected abattoir at market weight of 125 kg and approximately 200 days of age. Carcass collection procedures followed the guidelines set by National Pork Producers Council [13]. Hot carcass weight, including the head, was collected at the time of the harvest. After 24 hours of refrigeration at 4°C, carcass characteristics, such as the backfat depths at the 1st rib, 10th rib and last lumbar, as well as the longissimus muscle area (LMA), were measured. The *longissimus dorsi* (LD), collected from the right side loins, was packed and transported to the NCSU Processed Meat Laboratory, approximately an hour away from the abattoir, for storage at 2°C until further analysis.

### Pork quality measurements

The LD samples collected from the right side loins were cut into 2.54-cm thick chops and were used to measure the marbling score (1 to 10) and the color score (1 to 6). One hundred gram samples trimmed from each LD were placed on hooks and hung in a plastic bag at 2°C for 24 h to measure drip loss. The ultimate pH was determined by homogenizing a sample with a variable speed laboratory blender (Waring, New Hartford, CT, USA) and adding deionized water to dilute the sample at a 1:10 ratio. An Accumet Excel XL15 pH meter with glass tip probe (Thermo Fisher Scientific, Waltham, MA, USA) was used to determine the pH after blending the samples for 20 s. Objective color score Commission Internationale de l’Eclairage (CIE) L* (lightness), a* (redness), and b* (yellowness) was measured using a Minolta Chroma Meter (CR-200, Ramsey, NJ, USA). Using the D65 illuminant and 2° standard observer, which was calibrated with a standard white plate before each use, the color values were measured at three different positions on the surface of each chop and were averaged.

Slice shear force was conducted to estimate tenderness [14]. The frozen steaks were cut to 2.54 cm thick and weighed then thawed at 6°C for 24 h. Thawed steaks were weighed, and thawed temperature was measured. Steaks were cooked on a conveyorized impingement grill (1100 Series Impinger II Conveyorized Oven, Lincoln Foodservice, Cleveland, OH, USA). With the impingement grill operating at top heat = 210°C, bottom heat = 210°C steaks were cooked for approximately 12.5 min until an internal temperature of 70°C. After the steaks exited the belt grill, a needle thermocouple probe was inserted into the geometric center of the steak and post-cooking temperature rise was monitored with a hand-held thermometer (Fisher Scientific, Hampton, NH, USA). Cooked steaks were weighed. Steaks were sampled and slice shear force was measured as described by Shackelford et al [15] using a universal testing system (Instron model 5565, Canton, MA, USA).

### Sensory panel tests

Sensory panel tests were conducted at North Carolina State University (Raleigh, NC, USA). The first sensory panel test, which used the pork from Trial 1, had 110 consumer participants and the second test, which used the pork from Trial 2, had 101 consumer participants. The majority of the participants in both Trials, recruited through a screener launched to an on-line database maintained by the Sensory Service Center with over 3,000 members, were between the ages of 29 and 35 (Table 2). Subjects over the age of 18 and under 60 were recruited for this panel. At the end of each experiment, the participants were awarded a five-dollar grocery store gift card.

Upon arrival, the participants filled out an information sheet, answering questions related to their demographics and pork consumption characteristics. Then, the participants were given one sample at a time and were asked to indicate the overall liking as well as overall flavor, freshness and texture likings of the sample on a 9-point Hedonic scale for which 1 = dislike extremely and 9 = like extremely. Then the participants scored the texture and the moisture of the samples on a just about right (JAR) scale, where 1 or 2 = too little, 3 = JAR, and 4 or 5 = too much. Lastly, the participants indicated their purchase intent of the sample on a 5-point scale for which 1 or 2 = probably would not buy, 3 = maybe would or maybe would not buy, and 4 or 5 = probably would buy. The samples were prepared by thawing the LD at 4°C and cooking at 200°C on an Impinger conveyor oven. The cooked samples were wrapped, labeled, and kept warm at 75°C inside a warming cabinet. Before serving, the samples were cut into 2×2×1 cm^3^ cubes.

### Data analyses

For pork characteristics and sensory panel data, trial and sex were included in the statistical model as fixed effects using PROC general linear model in SAS 9.3. The interaction between trial and sex was excluded because it was not significant (p>0.05). Hot carcass weight was included in the model as a covariate for backfat thickness.

## RESULTS AND DISCUSSION

Presented in Table 1 are the least square means and standard errors of the meat quality traits by trials and sex. Pork from pigs in Trial 1 was significantly more yellow than the pork from pigs in Trial 2 (p<0.05). Also, pork from pigs in Trial 1 had significantly higher values for the drip loss and the slice shear force (p<0.05). Neither the subjective nor the objective CIE L*, a*, and b* color scores displayed any differences between the boars and the gilts (p>0.05). No difference was found for pH and marbling score between trials or sexes. As would be expected, gilts had a thicker backfat measurement at the last lumbar and a narrower LMA measurement when compared to the boars (p<0.05). The only difference in the sensory characteristics was found between the trials for texture and moisture JAR scores (Table 3). Pork from pigs in Trial 1 scored significantly higher in both the texture and moisture JAR Scores (p<0.05).

### Carcass and meat quality

In general, lean pork is preferred over fatty pork. When consumers from 23 different countries were presented with pork chops of varying fat contents, all but two countries, namely Korea and Japan, showed preference for lean meat, while no country showed a strong preference for the fatty meat [16]. Present study found significant difference in backfat and LMA measurements between gilts and uncastrated boars. The thinner backfat and larger LMA of the boars indicate leaner meat. These findings are consistent with those of the meta-analysis of the sex effect on pork quality performed by Trefan et al [17]. When intact/entire (uncastrated) males (EM), surgically castrated males (CM) and gilts (G) were compared, EM had the thinnest backfat measurement and the smallest intramuscular fat (IMF) percentage. Similar findings were reported by Grela et al [18]. When the EM, CM, and G of the Polish native Pulawska breed reared outdoors were compared. The EM had the thinnest backfat at the midback and the shoulder. Though the difference in LMA between EM and G were not significant, EM had slightly narrower LMA. Compared to the LMA of the CM, LMA of EM were significantly wider (p<0.05).

### Selective breeding as an alternative to surgical castration

Consumer preference for lean pork makes boar meat profitable, if boar taint weren’t an issue. For centuries, farmers have surgically castrated the male pigs as a way of preventing boar taint. However, growing consumer interest in animal welfare is bringing an end to the practice. In 2010, stakeholders from the European pig production industry voluntarily signed the ‘Brussels Declaration’ to eliminate the surgical castration of pigs without anesthesia or analgesia by 2012 and phase out surgical castration completely by 2018. These goals have not been met, however, due to the lack of a sufficient alternative method to prevent boar taint [19].

Currently, there are three major alternatives to surgical castration: immunocastration, genomic selection and breeding. Immunocastration is vaccination against gonadotropin-releasing hormone (GnRH). Vaccination against GnRH helps prevent boar taint by reducing the concentrations of testicular steroids, including androstenone and skatole [20]. Adaptation of immunocastration among farmers is low. Farmers showed concerns for consumer acceptability of the vaccination and were reluctant to readily apply immunocastration, referring to possible meat safety risks during consumption [21]. The notion of meat safety concerns was shared by the consumers as well. Given brief descriptions of immunocastration, consumers showed low acceptance of meat from immunocastrated animals [22]. Application of immunocastration in place of surgical castration seems unlikely unless consumer concerns regarding residues from the vaccination is resolved [23].

Genomic selection and breeding against boar taint are based on the idea of genetic modification that has long been practiced since the first domestication of livestock. Wild animals were domesticated by selectively breeding animals with high docility. Then, domesticated animals with favorable qualities, such as large litter size and faster growth, were selectively bred, resulting in genetically modified farm animals with high economic values. Recent advancements in technology have allowed for more accurate selection based on genetic data. The moderate-high heritability of the biochemical compounds responsible for boar taint makes genetic selection against boar taint based on these compounds possible [10]. While genomic selection may represent a long-term solution to the issue [24], extensive field applications in commercial pig population may be limited by the economic aspects associated with genomic selection. According to Samore, the price of genotyping, along with the increased expenses in additional infrastructure associated with genotyping, is too high compared to the value of the individual animals [25].

According to Backus et al [26], significant genetic variations of androstenone and skatole indicate heritable differences between breeds and families within breeds, and studies have found breed differences in the levels of boar taint compounds. When Belgian Landrace (BL), Large White (LW) and Pietrain (P) breeds were compared, P had the lowest levels of Skatole all throughout the varying slaughter weights (p<0.01), and the levels of androstenone were generally lower for both BL and P compared to LW except at the 90 kg slaughter weight (p<0.05) [8]. When levels of Skatole were compared between Yorkshire, Landrace, Hampshire and Duroc pigs, Duroc had the highest level with a left skewed frequency distribution of plasma skatole concentrations [27]. The breed differences in the levels of boar taint compounds suggest it is possible to selectively breed pigs that do not have boar taint. Babol et al [27] suggests that rearing environments may largely influence the variations in the levels of boar taint compounds. Present study found an elimination of boar taint in pork from organically reared HB crossbreds. This study is the first to evaluate the sensory characteristics of organically reared HB crossbreds, and thus direct comparisons are not feasible. However, a number of other studies have also related rearing conditions with the amount of boar taint compounds in the meat. van Wagenberg et al [28] found an association between smaller group size and lower farm-level boar taint prevalence. Along with findings by Giersing et al [29], which showed that the aggressive behavior of boars is associated with the level of androstenone in the pigs, the association between smaller group size and lower farm-level boar taint prevalence helps explain the elimination of boar taint found in the present study. According to Cornale et al [30], pigs housed in low stocking density with environmental enrichment show less aggressive behavior. This suggests that rearing pigs organically, in low stocking density with access to straw bedding or other forms of enrichment, reduces the aggressive behavior of boars and thus reduces the chance of tainted meat.

## CONCLUSION

In these Berkshire crossbreds pigs sired by Hereford boars reared outdoors, the gilts had thicker backfat measurements at the last lumbar but narrower loin muscle areas (p<0.05). However, no difference in the IMF content, or marbling, was observed (p>0.05). Furthermore, no difference in the eating quality of pork was observed between the sexes (p>0.05). Consumers were not able to detect boar taint in these intact males reared outdoors and slaughtered at market weight. However, based on this and other studies, the effects of sex seem to differ with the breed studied. Additional trials would be necessary; however, based on the results of the present study, outdoor rearing can be suggested as a solution to the issue of boar taint.

## Figures and Tables

**Table 1:** Least square means and standard errors of each characteristic by trial and sex for pigs raised in alternative production system

Variables measured	Trial		Sex	
	
	1	2	Boar	Gilt
pH	5.76±0.02	5.73±0.03	5.76±0.03	5.73±0.03
Color score	3.44±0.19	3.20±0.24	3.59±0.22	3.05±0.22
L*	53.2±0.92	54.5±1.16	53.4±1.03	54.3±1.03
a*	9.19±0.44	8.04±0.56	9.18±0.50	8.05±0.50
b*	6.03±0.44^a^	4.32±0.56^b^	5.40±0.50	4.95±0.50
Marbling score	2.31±0.29	1.60±0.37	1.76±0.33	2.15±0.33
BF1 (cm)	4.35±0.22	4.37±0.29	4.42±0.25	4.30±0.24
BF10 (cm)	1.78±0.07	1.93±0.09	1.84±0.08	1.88±0.07
BFLL (cm)	2.63±0.19	2.95±0.25	2.24±0.21^a^	3.35±0.20^b^
LMA (cm^2^)	50.8±1.68	47.2±2.20	51.8±1.84^a^	46.3±1.79^b^
Drip loss (%)	2.64±0.29^a^	1.36±0.36^b^	2.15±0.32	1.85±0.32
Slice shear force	19.0±0.80^a^	15.8±1.01^b^	17.4±0.90	17.5±0.90

L*, indicates muscle lightness; a*, a measure of muscle redness; b*, a measure of muscle yellowness; BF1, 1st rib backfat; BF10, 10th rib backfat; BFLL, last lumber backfat; LMA, loin muscle area.

a,b Means with different superscripts among groups within each variables measured differ at p<0.05.

**Table 2:** Demographic information and consumer consumption characteristics for sensory tests on pork chops from pigs raised in an outdoor hoop barn

Consumer consumption characteristics	Demographic information	Trial 1 (n = 110; %)	Trial 2 (n = 101; %)
Gender	Male	45.0	49.5
	Female	65.0	50.5
Age	18 years old or younger	0.0	0.0
	19–24 years old	12.7	26.7
	25–35 years old	38.2	36.6
	36–45 years old	14.5	8.9
	46–55 years old	17.3	16.9
	56–65 years old	15.5	10.9
	66–70 years old	0.9	0.0
	71 years old and over	0.9	0.0
Primary shopper	Yes	93.6	88.1
	No	6.4	11.9
Purchased and consumed	Today	2.7	2.0
	At least once within the last week	26.4	30.7
	At least once within the last two weeks	23.6	24.8
	At least once within the last month	23.6	15.8
	At least once within the last 2 months	8.2	7.9
	At least once within the last 3 months	3.6	9.9
	At least once within the last 6 months	8.2	5.9
	At least once within the last year	0.9	3.0
	I do not purchase pork chops	2.8	0.0
Future purchase and consume	Yes	99.1	100.0
	No	0.9	0.0
Income	<$19,999 per year	8.2	12.9
	$20,000 – $29,999 per year	5.5	12.9
	$30,000 – $39,999 per year	15.5	5.9
	$40,000 – $49,999 per year	16.4	17.8
	$50,000 – $59,999 per year	8.2	12.9
	$60,000 – $69,999 per year	12.7	8.9
	$70,000 – $79,999 per year	7.3	7.9
	$80,000 – $89,999 per year	10.0	5.0
	Greater than $90,000 per year	16.2	15.8

* Percentage of consumers that selected these options is presented and provides a sum total of 100% for each category.

**Table 3:** Least square means and standard errors of sensory traits evaluating meat from Berkshire crossbreds sired by Hereford boars

Sensory traits	Trial		Sex	
	
	1	2	Male	Female
Overall liking1)	5.77±0.12	5.97±0.12	6.01±0.12	5.73±0.12
Overall flavor liking1)	5.87±0.11	5.93±0.12	5.99±0.12	5.82±0.12
Freshness liking1)	6.17±0.10	6.20±0.11	6.28±0.10	6.09±0.10
Texture liking1)	5.38±0.13	5.71±0.14	5.66±0.14	5.43±0.14
Texture JAR2)	3.64±0.05^a^	3.50±0.05^b^	3.56±0.05	3.57±0.05
Moisture JAR2)	2.56±0.04^a^	2.75±0.04^b^	2.71±0.04	2.60±0.04
Purchase intent3)	2.94±0.08	3.00±0.09	3.06±0.08	2.86±0.08

1) Liking attributes are scored on a 9-point hedonic scale where 1 = dislike extremely and 9 = like extremely.

2) Just about right (JAR) scales are scored on a 5-point scale where too little = 1 or 2, JAR =3 and too much = 4 or 5.

3) Purchase intent questions were scored on a 5pt scale where 1 or 2 = probably would not buy, 3 = maybe would or maybe would not buy, and 4 or 5 = probably would buy.

a,b Least square means with different superscripts differ (p<0.05).
